# Microbial diversity and functional analysis in wastewater and sludge of wastewater treatment plants

**DOI:** 10.7717/peerj.21546

**Published:** 2026-07-16

**Authors:** Yazi Li, Shuhong Zhang, Ke Xu, Jingjie Zhang, Man Dong, Huan Lu, Yongshan Fan

**Affiliations:** 1 Department of Life Sciences, Hebei Key Laboratory of Plant Biotechnology Research and Application, Tangshan Normal University, Tangshan, Hebei, China; 2 College of Plant Protection, Yunnan Agricultural University, Kunming, Yunnan, China; 3 College of Life Sciences, Agricultural University of Hebei, Baoding, Hebei, China

**Keywords:** Wastewater treatment plant, Bacteria, Fungi, Diversity, Functional prediction

## Abstract

**Background:**

The efficiency of wastewater treatment plants (WWTPs) relies heavily on microbial communities. However, the microbial characteristics of different treatment units in the Qian’an WWTP (Hebei, China) remain unclear. This study investigates its microbial diversity and functions to provide a basis for process optimization.

**Methods:**

Samples were collected in October 2024 from four representative units were selected: sludge (group A), sedimentation tank water (group C), aeration tank water (group E), and raw wastewater (group F). Bacterial and fungal communities were analyzed *via* Illumina NextSeq 2000 PE300 platform sequencing, with functional potentials predicted using PICRUSt2 and FUNGuild, respectively.

**Results:**

Bacterial richness was highest in groups A, C, and E and lowest in group F, whereas fungal richness was highest in groups C and E and lowest in group F. The microbial community structures of groups C and E were highly similar in terms of richness and diversity patterns, but both differed markedly from groups A and F. At the phylum level, bacteria in group A were significantly enriched in Chloroflexi and Firmicutes, and fungi by Rozellomycota; bacteria in groups C, E and F were mainly Proteobacteria and Bacteroidetes. Fungal composition varied significantly, with Rozellomycota in A, Blastocladiomycota in C/E, and Ascomycota in F. Predicted Kyoto Encyclopedia of Genes and Genomes (KEGG) analysis revealed 25 differential metabolic pathways (*e.g*., amino acid and carbohydrate metabolism). Furthermore, 24 predicted functional genes related to nitrogen metabolism (nitrification, nitrogen fixation) were inferred, suggesting strong nitrogen cycling potential. Fungal functional groups differed significantly among groups: Ectomycorrhizal fungi dominated in group A, Insect Parasite-Undefined Saprotroph in groups C and E, and Animal Pathogen was predicted to account for up to 54.41 ± 4.75% in group F.

**Conclusions:**

This study clarifies the microbial characteristics across treatment units and highlights the nitrogen cycling potential of the WWTP microbiome, providing a scientific basis for optimizing treatment processes.

## Introduction

With the rapid advancement of industrialization and urbanization, the discharge of domestic sewage and industrial wastewater has kept rising, and water pollution has become one of the key challenges restricting the sustainable development of the ecological environment (Strathmann et al., 2016). As the core infrastructure for water environmental protection, the purification efficiency of wastewater treatment plants (WWTPs) directly determines the pollutant removal rate and the safety of effluent quality (de Celis et al., 2020). As the “core driving” force of wastewater treatment systems, microbial communities dominate key purification processes such as organic pollutant decomposition and nitrogen and phosphorus cycling through physiological processes including metabolism, degradation, and transformation (Begmatov et al., 2022; Tang & Zhao, 2025). Therefore, revealing the structural characteristics, diversity differences, and functional potential of microbial communities in wastewater treatment systems provides a scientific basis for optimizing process parameters, improving pollutant removal efficiency, and ensuring the stable operation of wastewater treatment systems.

As a typical regional treatment system for mixed industrial and domestic wastewater, the wastewater treatment system of Qian’an Wastewater Treatment Plant is responsible for purifying industrial effluent and part of the surrounding domestic sewage. The core biological treatment units such as sludge and aeration tank water in this system show obvious differentiation in microbial community structure due to significant differences in influent pollutant composition and dissolved oxygen level, thus forming unique microbial ecological niches with function-specific microorganisms in each unit (Zhang, Xu & Zhu, 2017). However, the microbial community composition, diversity distribution and functional metabolic characteristics of different treatment units in this wastewater treatment plant are still unclear at present; it remains unknown whether the microbial community structure in sludge, as a “hotspot” for pollutant accumulation and microbial colonization, is significantly different from that in water units (sedimentation tank and aeration tank), and what the interaction is between exogenous microorganisms carried by raw wastewater and the domesticated microbial communities in the treatment units. More importantly, whether there are targeted differences in the functional potential of microbial communities in different units (such as genes related to nitrogen metabolism and pollutant degradation) and whether they can provide targets for process optimization are the key scientific questions, and answering these questions is of urgent practical significance for improving nitrogen removal efficiency and reducing effluent pollutant concentration in this wastewater treatment plant.

In recent years, high-throughput sequencing technologies (*e.g*., 16S rRNA gene and Internal Transcribed Spacer (ITS) sequencing) combined with bioinformatics analyses (*e.g*., PICRUSt2 functional prediction and FUNGuild ecological functional annotation) have become mainstream approaches for dissecting environmental microbial communities (Di Bella et al., 2013; Douglas et al., 2020). These technologies enable the rapid profiling of microbial taxa and the prediction of functional potential, accurately revealing the mechanisms linking community structure to environmental factors. Accordingly, this study aims to clarify the diversity differences of bacterial and fungal communities in different treatment units of the Qian’an Wastewater Treatment Plant and reveal the spatial distribution pattern of community structure, analyze the compositional characteristics of dominant microbial groups at the phylum and genus levels and identify the representative microbial taxa in each unit, predict the functional metabolic pathways and ecological functions of microorganisms in different units and explore key functional genes and functional groups, and ultimately provide a scientific basis and technical reference for optimizing the wastewater treatment process and improving nitrogen removal efficiency of the plant based on microbial community characteristics and functional potential. The findings of this study can not only fill the research gap in microbial ecology of the Qian’an Wastewater Treatment Plant, but also provide theoretical support for microbial regulation and process upgrading of wastewater treatment systems.

## Materials and Methods

### Study area and sample collection

Samples were collected in October 2024 from the wastewater treatment plant in Qian’an, including sludge (group A: A1, A2, A3), sedimentation tank water (group C: C1, C2, C3), aeration tank water (group E: E1, E2, E3) and raw wastewater (group F: F1, F2, F3). Three 2 L homogeneous water samples were collected at each sampling site. Meanwhile, sludge samples were collected with a sterile sampling shovel, transferred into pre-sterilized 50 mL centrifuge tubes, stored in an ice box at low temperature, and transported back to the laboratory immediately.

### DNA extraction and high-throughput sequencing

Genomic DNA was extracted from all samples using the E.Z.N.A^™^ Mag-Bind Soil DNA Kit (Omega Bio-tek, Norcross, GA, USA). The hypervariable V3-V4 region of the bacterial 16S rRNA gene was amplified by polymerase chain reaction (PCR) with the universal primers 341F (5′-CCTAYGGGRBGCASCAG-3′) and 806R (5′-GGACTACNNGGGTATCTAAT-3′). The ITS3-ITS4 region of the fungal ITS gene was amplified with the universal primers ITS3 (5′-GATGAAGAACGYAGYRAA-3′) and ITS4 (5′-TCCTCCGCTTATTGATATGC-3′) (Ni et al., 2025; Op De Beeck et al., 2014). Agarose gel electrophoresis was used to detect the purity and fragment size of PCR products, and a Qubit 3.0 fluorometer was applied to quantify the library concentration. All libraries were mixed equally at a 1:1 ratio. The purified amplicons were sequenced on the Illumina NextSeq 2000 PE300 platform at Sangon Biotech (Shanghai) Co., Ltd. (Shanghai, China). Three biological replicates were set for each sample.

### Data processing

The data processing workflow for samples including sludge (group A: A1, A2, A3), sedimentation tank water (group C: C1, C2, C3), aeration tank water (group E: E1, E2, E3) and raw wastewater (group F: F1, F2, F3) was as follows: after obtaining the sequencing data of all samples, cutadapt 1.1.8 software was used to identify and remove primer adapter sequences. Then, paired-end (PE) reads were merged into a single sequence using PEAR v0.9.8 based on the overlap relationship between PE reads. Subsequently, sample-specific data were identified and distinguished according to barcode tag sequences. Finally, PRINSEQ v0.20.4 was used to truncate bases with a quality score below 20 at the tail of reads to obtain valid data for each sample.

Taxonomic assignment was performed using specific algorithms for each marker: 16S rRNA sequences were classified against the silva v138.1 database using the Ribosomal Database Project (RDP) classifier v2.12 with a Naive Bayesian algorithm, while ITS sequences were compared against the UNITE v9.0 database using BLAST+ v2.10.0. Specifically, prior to taxonomic assignment, Operational Taxonomic Unit (OTU) clustering at a 97% sequence similarity threshold and taxa abundance tables were generated using USEARCH v11.0.667. The barplot function in R v3.6.0 (R Core Team, 2019) was employed to visualize the community structure diagrams of the samples at each taxonomic level. Additionally, the principal component analysis (PCA) plot was generated using the ggscatter function from the ggpubr package in R v3.6.0. The vegan package was used for multivariate statistical analysis. Mothur v1.43.0 software was used to evaluate the αlpha diversity indices of samples (Schloss et al., 2009). PICRUSt2 v2.5.2 software was employed to predict the functional abundance of bacterial samples based on the sequence abundance of the 16S rRNA gene, while FUNGuild v1.0 software was used to predict the functional abundance of fungal samples based on the sequence abundance of the ITS gene (Li et al., 2025; Carles et al., 2021). Raw sequencing data have been deposited in the National Microbiology Data Center (NMDC), and detailed sample metadata linking the sample IDs used in this study to NMDC accession numbers are provided in Table S1.

### Statistical analysis

Mothur 1.43.0 software was used to calculate the Alpha diversity indices of samples, including observed taxa (ACE), Chao index, Simpson index and Shannon index (Schloss et al., 2009). Excel 2010 was applied to analyze and process the data, and to generate corresponding tables. One-way analysis of variance (ANOVA) with Tukey’s multiple comparisons test was performed using GraphPad Prism 8.0 to compare alpha diversity indices, microbial community structure and fungal functional predictions among the four groups. Differential abundances of Kyoto Encyclopedia of Genes and Genomes (KEGG) pathways and Clusters of Orthologous Groups (COG) functional categories were analyzed using STAMP 2.1.3 software. Pairwise comparisons between groups were conducted using Welch’s t-test. Differences in all statistical tests were evaluated using adjusted *P* values corrected for multiple comparisons, and adjusted *P* < 0.05 was considered statistically significant.

## Results

### Statistics of valid data from sample sequencing

**Figure 1:**
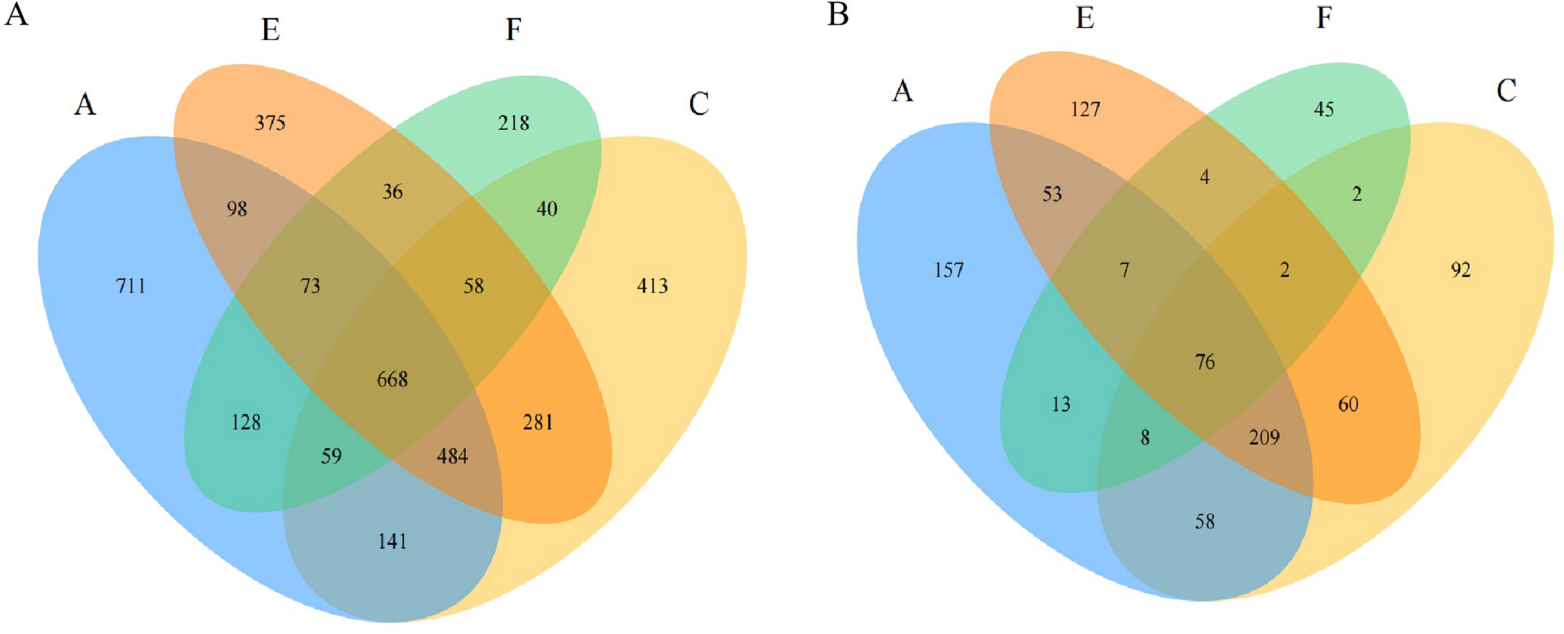
Venn diagram of bacterial and fungal sample distribution in wastewater treatment plant. Different samples (groups) are displayed in different colors. The numbers in the figure represent the number of unique or shared taxa (taking OTU level as an example). Sludge (A: A1, A2, A3), sedimentation tank water (C: C1, C2, C3), aeration tank water (E: E1, E2, E3) and raw water (F: F1, F2, F3).

Raw sequencing data have been deposited in the National Microbiology Data Center (NMDC), with detailed sample metadata provided in Table S1. Following sequencing of bacteria and fungi from wastewater treatment plant sludge (A: A1, A2, A3), sedimentation tank water (C: C1, C2, C3), aeration tank water (E: E1, E2, E3), and raw water (F: F1, F2, F3), the detection results for each sample were obtained by barcode identification. A total of 199,654 valid sequences were obtained from sludge bacterial samples, with an average of 66,551 valid sequences per sample; 190,427 valid sequences were obtained from sedimentation tank water bacterial samples, with an average of 63,476 valid sequences per sample; 177,026 valid sequences were obtained from aeration tank bacterial samples, with an average of 59,009 valid sequences per sample; and 186,775 valid sequences were obtained from raw water bacterial samples, with an average of 62,258 valid sequences per sample (Table S2). There were 1,323 shared OTUs between sludge bacterial samples and aeration tank bacterial samples, 928 shared OTUs between sludge bacterial samples and raw water bacterial samples, 1,352 shared OTUs between sludge bacterial samples and sedimentation tank water bacterial samples, 835 shared OTUs between aeration tank bacterial samples and raw water bacterial samples, 1,491 shared OTUs between aeration tank bacterial samples and sedimentation tank water bacterial samples, and 825 shared OTUs between raw water bacterial samples and sedimentation tank water bacterial samples. At a 97% sequence similarity threshold, a total of 668 OTUs were shared by bacterial samples from sludge, sedimentation tank water, aeration tank water, and raw water (Fig. 1A).

A total of 443,197 valid sequences were obtained from fungal samples of sludge (A: A1, A2, A3), with an average of 147,732 valid sequences per sample. A total of 362,978 valid sequences were obtained from fungal samples of sedimentation tank water (C: C1, C2, C3), with an average of 120,993 valid sequences per sample. A total of 368,039 valid sequences were obtained from fungal samples of aeration tank water (E: E1, E2, E3), with an average of 122,680 valid sequences per sample. A total of 436,883 valid sequences were obtained from fungal samples of raw water (F: F1, F2, F3), with an average of 145,628 valid sequences per sample (Table S3). There were 345 shared OTUs between sludge fungal samples and aeration tank fungal samples, 104 shared OTUs between sludge fungal samples and raw water fungal samples, 351 shared OTUs between sludge fungal samples and sedimentation tank water fungal samples, 89 shared OTUs between aeration tank fungal samples and raw water fungal samples, 347 shared OTUs between aeration tank fungal samples and sedimentation tank water fungal samples, and 88 shared OTUs between raw water fungal samples and sedimentation tank water fungal samples. In addition, a total of 76 OTUs were shared by fungal samples from sludge, sedimentation tank water, aeration tank water and raw water (Fig. 1B).

### Alpha diversity analysis

Alpha diversity was mainly reflected by six indices, namely Simpson, Chao, ACE, Shannon, Shannoneven and Coverage, which characterize taxa richness and evenness.

Coverage refers to the microbial coverage rate. The higher the value, the lower the probability that new taxa in the sample were not detected.

For bacterial communities from sludge (A: A1, A2, A3), sedimentation tank water (C: C1, C2, C3), aeration tank water (E: E1, E2, E3) and raw water (F: F1, F2, F3), the Chao and ACE richness indices showed a sequential decreasing trend from sludge to raw water (Table 1; Additional file 1 Table S1). *Post-hoc* Tukey’s multiple comparisons (after one-way ANOVA) revealed no significant differences in Chao and ACE values among groups A, C and E (adjusted *P* > 0.05). For the Chao index, groups A, C and E exhibited extremely significantly higher richness than raw water group F (adjusted *P* < 0.0001). For the ACE index, group A was significantly higher than F (adjusted *P* < 0.01), while groups C and E were significantly higher than F (adjusted *P* < 0.05). In addition, the Coverage index of all samples was 0.99, close to 1, indicating that the sequencing results are reliable (Table 1). The rarefaction curves further confirm this conclusion (Fig. S1A).

**Table 1:** Statistics of alpha diversity indices for bacterial samples from wastewater treatment plant.

Index	Group	Shannon	Chao	Ace	Simpson	Shannoneven	Coverage
Average ± SD	A	4.79 ± 0.02b	1,772.52 ± 41.76a	1,864.76 ± 48.16a	0.03 ± 0.00b	0.65 ± 0.00b	0.99 ± 0.00a
Average ± SD	C	5.15 ± 0.11a	1,736.02 ± 64.41a	1,793.5 ± 94.64a	0.02 ± 0.00c	0.71 ± 0.02a	0.99 ± 0.00a
Average ± SD	E	5.08 ± 0.05a	1,634.78 ± 18.39a	1,718.01 ± 32.21a	0.02 ± 0.00bc	0.7 ± 0.01ab	0.99 ± 0.00a
Average ± SD	F	2.26 ± 0.03c	1,022.64 ± 143.98b	1,266.26 ± 275.68b	0.24 ± 0.00a	0.34 ± 0.01c	0.99 ± 0.00a

**Notes:**

Sludge (A: A1, A2, A3), sedimentation tank water (C: C1, C2, C3), aeration tank water (E: E1, E2, E3) and raw water (F: F1, F2, F3).

Different lowercase letters indicate significant differences between groups (Tukey’s test, *P* < 0.05).

For fungal communities across sludge (A), sedimentation tank water (C), aeration tank water (E) and raw water (F), Chao richness ranked C > E > A > F and ACE ranked E > C > A > F (Table 2; Additional file 2 Table S2). No significant differences existed among A, C and E (adjusted *P* > 0.05). A, C and E were significantly richer than F for Chao (adjusted *P* < 0.05); for ACE, C (adjusted *P* < 0.01) and E (adjusted *P* < 0.05) were significantly higher than F, while A and F showed no difference (adjusted *P* > 0.05). Shannon diversity and Shannoneven evenness followed A > C > E > F, and Simpson dominance followed F > E > C > A. Group A differed significantly from C, E and F (adjusted *P* < 0.0001). No differences were found between C and E (adjusted *P* > 0.05). C and E showed no significant Shannon difference from F (adjusted *P* > 0.05), but differed markedly in Simpson and Shannoneven indices (adjusted *P* < 0.01–0.001). In addition, the Coverage index of the fungal samples was close to 1, indicating that the sequencing results were reliable (Table 2). The rarefaction curves further confirm this conclusion (Fig. S1B).

**Table 2:** Statistics of alpha diversity indices of fungal samples in wastewater treatment plants.

Index	Group	Shannon	Chao	Ace	Simpson	Shannoneven	Coverage
Average ± SD	A	3.24 ± 0.19a	381.34 ± 180.32a	386.38 ± 186.05ab	0.09 ± 0.02c	0.57 ± 0.02a	1.00 ± 0.00a
Average ± SD	C	2.14 ± 0.03b	453.99 ± 62.47a	542.46 ± 90.09a	0.22 ± 0.01b	0.37 ± 0.01c	1.00 ± 0.00a
Average ± SD	E	2.10 ± 0.06b	453.50 ± 58.52a	500.53 ± 85.38a	0.24 ± 0.02b	0.36 ± 0.01c	1.00 ± 0.00a
Average ± SD	F	1.95 ± 0.19b	105.42 ± 63.71b	105.41 ± 38.6b	0.32 ± 0.02a	0.45 ± 0.01b	1.00 ± 0.00a

**Notes:**

Sludge (A: A1, A2, A3), sedimentation tank water (C: C1, C2, C3), aeration tank water (E: E1, E2, E3) and raw water (F: F1, F2, F3).

Different lowercase letters indicate significant differences between groups (Tukey’s test, *P* < 0.05).

### Beta diversity analysis

Principal components analysis (PCA) was conducted based on Bray-Curtis distances, and the principal coordinate combination with the highest contribution rate was selected for plotting and visualization. Differences between sample groups were observed *via* PCA. In the results, different colors represent different groups. The closer the distance between samples, the more similar the microbial community composition and structure; conversely, the greater the difference.

**Figure 2:**
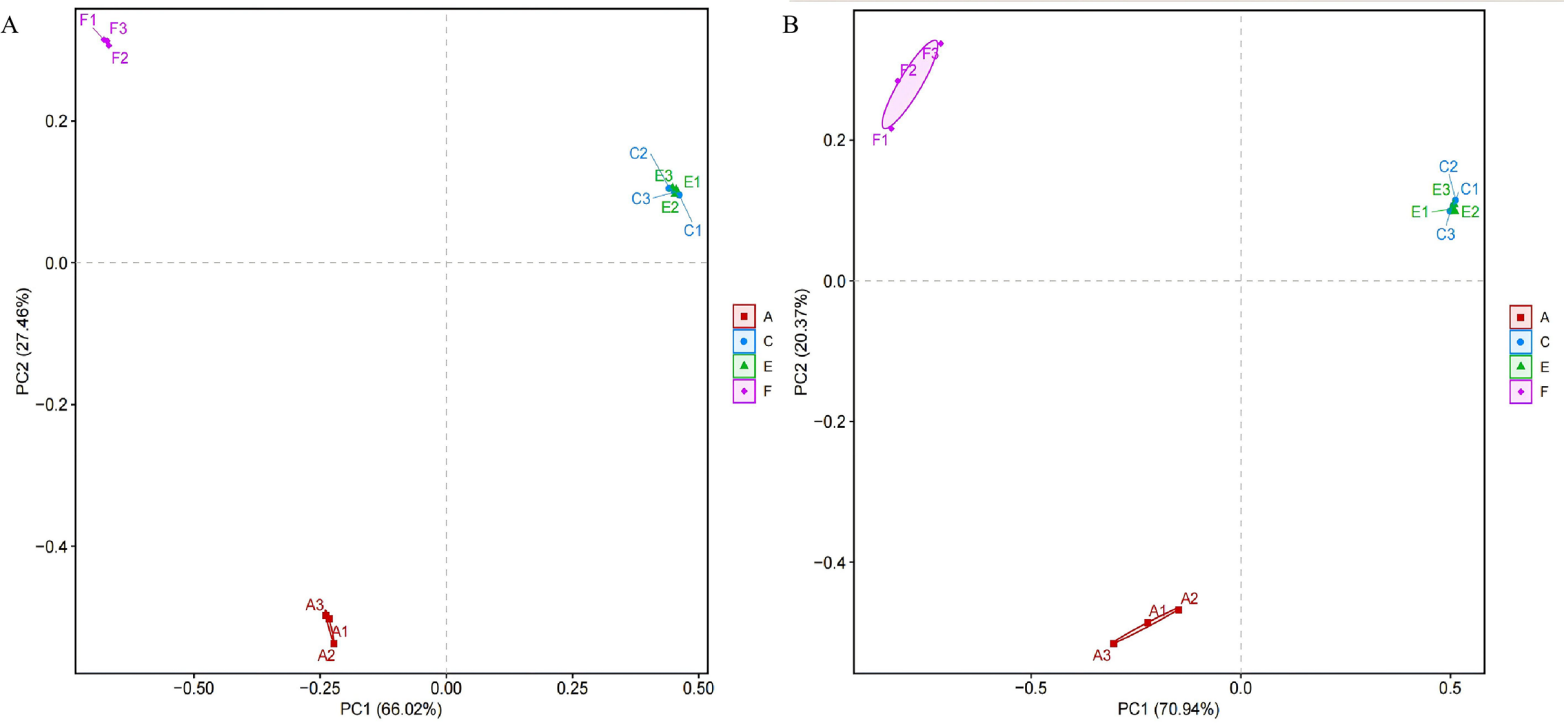
PCA plot of bacterial and fungal samples in wastewater treatment plants. The horizontal and vertical axes represent the two selected principal component axes, and the percentages indicate the interpretation degrees of the principal components for differences in sample composition. The scales of the horizontal and vertical axes are relative distances with no actual significance. Points with different colors or shapes represent samples from different groups. The closer two sample points are, the more similar the taxa composition between the two samples (at the OTU level). Sludge (A: A1, A2, A3), sedimentation tank water (C: C1, C2, C3), aeration tank water (E: E1, E2, E3) and raw water (F: F1, F2, F3).

The bacterial and fungal samples of sedimentation tank water (C: C1, C2, C3) and aeration tank water (E: E1, E2, E3) from Qian’an Wastewater Treatment Plant in Tangshan City were both closely distributed/clustered, indicating small differences in their respective bacterial and fungal community compositions. Meanwhile, these water-borne bacterial and fungal samples were all far from the bacterial and fungal sample groups of sludge (A: A1, A2, A3) and raw water (F: F1, F2, F3), revealing clear differences in bacterial and fungal community compositions between water and sludge/raw water samples (Figs. 2A, 2B).

### Taxonomic composition analysis

#### Microbial community composition at the phylum level

At the phylum level, 15 dominant taxa were selected based on taxa composition analysis of bacterial samples from sludge (A: A1, A2, A3), sedimentation tank water (C: C1, C2, C3), aeration tank water (E: E1, E2, E3) and raw water (F: F1, F2, F3), and presented in the form of stacked bar charts of relative taxa abundance for each group.

**Figure 3:**
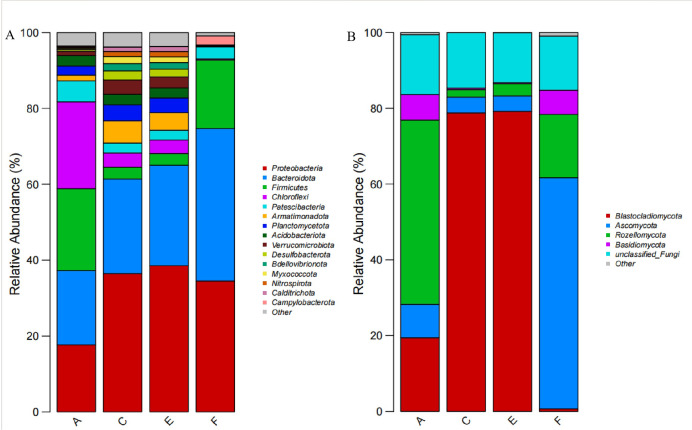
Relative abundance bar chart of bacterial and fungal samples at the phylum level in wastewater treatment plant. (A) Relative abundance bar chart of bacterial samples at the phylum level in wastewater treatment plant; (B) Relative abundance bar chart of fungal samples at the phylum level in wastewater; Sludge (A: A1, A2, A3), sedimentation tank water (C: C1, C2, C3), aeration tank water (E: E1, E2, E3) and raw water (F: F1, F2, F3).

Among them, the dominant bacterial phyla in sludge samples were Chloroflexi (22.77 ± 4.77%), Firmicutes (21.68 ± 2.31%) and Bacteroidota (19.64 ± 1.23%). The dominant phyla in sedimentation tank water were Proteobacteria (36.47 ± 1.75%), Bacteroidota (24.90 ± 3.64%) and Armatimonadota (5.83 ± 2.29%). The dominant phyla in aeration tank water were Proteobacteria (38.58 ± 1.21%), Bacteroidota (26.47 ± 1.38%) and Armatimonadota (4.60 ± 0.43%). The dominant phyla in raw water were Bacteroidota (40.24 ± 3.45%), Proteobacteria (34.47 ± 3.62%) and Firmicutes (18.05 ± 0.20%) (Fig. 3A, Additional file 3 Table S3). Statistical analysis revealed that Chloroflexi and Firmicuteswere significantly enriched in sludge (group A) compared to groups C and E (adjusted *P* < 0.0001), and Firmicutesin group A was also significantly higher than in raw water (group F) (adjusted *P* < 0.05). Conversely, Bacteroidotawas significantly more abundant in raw water (group F) than in all other groups (adjusted *P* < 0.001), while Proteobacteria showed a different pattern: groups E and C were both significantly enriched compared to group A (adjusted *P* < 0.0001), with group E being the highest. Additionally, group F also had significantly higher abundance than group A (adjusted *P* < 0.0001), confirming the overall trend of E > C > F > A.

At the phylum level, five dominant taxa were selected based on taxa composition analysis of fungal samples from sludge (A: A1, A2, A3), sedimentation tank water (C: C1, C2, C3), aeration tank water (E: E1, E2, E3) and raw water (F: F1, F2, F3), and presented as stacked bar charts of relative taxa abundance for each group. Among them, the dominant fungal phyla in sludge samples were Rozellomycota (47.97 ± 7.08%), Blastocladiomycota (19.93 ± 8.24%) and unclassified_Fungi (15.80 ± 2.45%). The dominant phyla in sedimentation tank water were Blastocladiomycota (78.74 ± 1.14%), unclassified_Fungi (14.71 ± 0.88%) and Ascomycota (4.15 ± 0.09%). The dominant phyla in aeration tank water were Blastocladiomycota (79.16 ± 2.81%), unclassified_Fungi (13.12 ± 2.32%) and Ascomycota (4.08 ± 0.43%). The dominant phyla in raw water were Ascomycota (61.40 ± 6.12%), Rozellomycota (16.55 ± 3.09%) and unclassified_Fungi (13.91% ± 5.24%) (Fig. 3B, Additional file 4 Table S4). Statistical analysis revealed that Blastocladiomycotawas dominant in groups E and C compared to group F (adjusted *P* < 0.0001). Conversely, Ascomycotawas significantly enriched in raw water (group F) compared to group E (adjusted *P* < 0.0001).

#### Microbial community composition at the genus level

**Figure 4:**
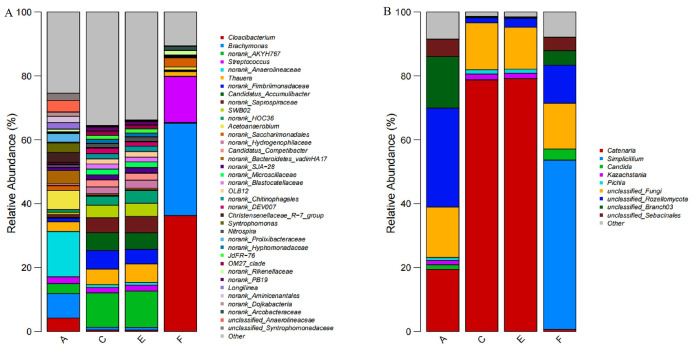
Relative abundance bar chart of bacterial and fungal samples at the genus level in wastewater treatment plant. (A) Relative abundance bar chart of bacterial samples at the genus level in wastewater treatment plant; (B) Relative abundance bar chart of fungal samples at the genus level in wastewater treatment plant; Sludge (A: A1, A2, A3), sedimentation tank water (C: C1, C2, C3), aeration tank water (E: E1, E2, E3) and raw water (F: F1, F2, F3).

At the genus level, 37 dominant taxa were selected based on taxa composition analysis of bacterial samples from sludge (A: A1, A2, A3), sedimentation tank water (C: C1, C2, C3), aeration tank water (E: E1, E2, E3) and raw water (F: F1, F2, F3), and presented as stacked bar charts of relative taxa abundance for each group (Fig. 4A, Additional file 5 Table S5). Among them, the dominant bacterial genera in sludge samples were *norank_Anaerolineaceae* (14.14 ± 2.74%) and *Brachymonas* (7.69 ± 1.05%). The dominant bacterial genera in sedimentation tank water samples were *norank_AKYH767* (10.72 ± 2.84%) and *norank_Fimbriimonadaceae* (5.82 ± 2.28%). The dominant bacterial genera in aeration tank water samples were *norank_AKYH767* (11.32 ± 1.08%) and *Thauera* (5.79 ± 0.34%). The dominant bacterial genera in raw water samples were *Cloacibacterium* (36.35 ± 3.36%), *Brachymonas* (28.85 ± 3.64%) and *Streptococcus* (14.39 ± 0.23%). For *Thauera*, the abundance distribution followed the trend E > C > A > F. Specifically, the aeration tank (group E) showed a significantly higher abundance than the sedimentation tank (group C, adjusted *P* < 0.05). Furthermore, both groups E and C were significantly enriched compared to the sludge (group A) and raw water (group F) (adjusted *P* < 0.001), while group A also exhibited a significantly higher abundance than group F (adjusted *P* < 0.001). In contrast, norank_Anaerolineaceae, the abundance was strictly confined to the sludge samples (group A), which exhibited a significantly higher abundance compared to all other groups (adjusted *P* < 0.0001). In contrast, no significant differences were observed among the liquid environments (groups C, E, and F, adjusted *P* > 0.05), following the distinct trend of A > C > E > F.

At the genus level, nine dominant taxa were selected based on taxa composition analysis of fungal samples from sludge (A: A1, A2, A3), sedimentation tank water (C: C1, C2, C3), aeration tank water (E: E1, E2, E3) and raw water (F: F2, F2, F3), and presented as stacked bar charts of relative taxa abundance for each group (Fig. 4B, Additional file 6 Table S6). Among them, the dominant fungal genera in sludge samples were unclassified_*Rozellomycota* (30.60 ± 4.11%), *Catenaria* (19.93 ± 8.23%) and unclassified_Fungi (15.80 ± 2.45%). The dominant fungal genera in sedimentation tank water samples were *Catenaria* (78.74 ± 1.14%), unclassified_Fungi (14.71 ± 0.88%) and *Kazachstania* (1.75 ± 0.05%). The dominant fungal genera in aeration tank water samples were *Catenaria* (79.16 ± 2.81%), unclassified_Fungi (13.12 ± 2.32%) and *Kazachstania* (1.65 ± 0.15%). The dominant fungal genera in raw water samples were *Simplicillium* (53.20 ± 4.09%), unclassified_Fungi (13.91 ± 5.24%) and unclassified_*Rozellomycota* (11.73 ± 3.69%). Statistical analysis revealed that *Catenaria* was the absolute dominant genus in groups C and E, with no significant difference between them (adjusted *P* > 0.05). Both groups C and E were significantly higher than groups A and F (adjusted *P* < 0.0001), while group F also showed a significantly higher abundance than group A (adjusted *P* < 0.01).

### Functional gene prediction and annotation analysis

#### KEGG function prediction analysis

**Figure 5:**
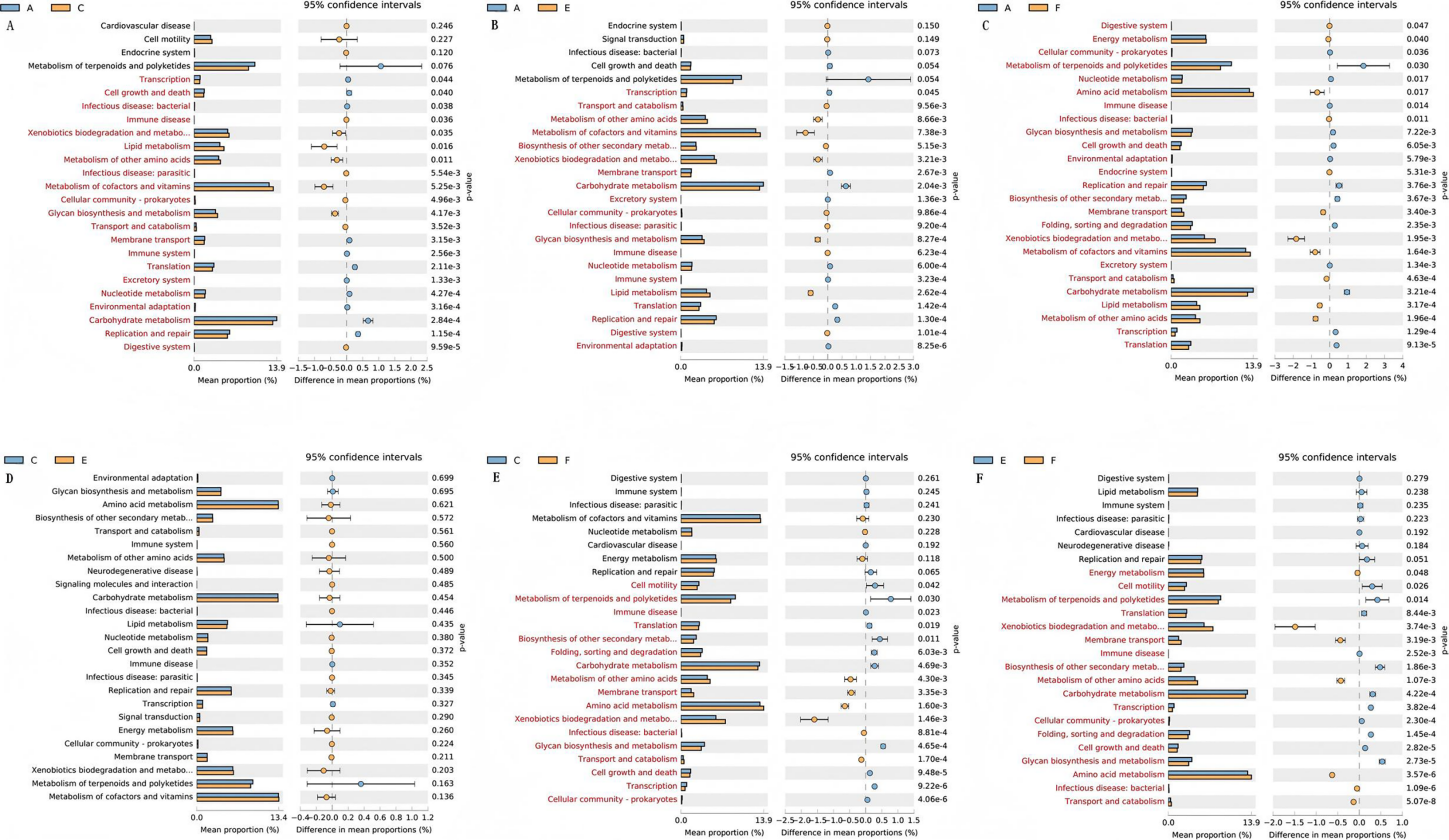
Differential analysis of KEGG metabolic pathways in bacterial communities between sludge (A), sedimentation tank water (C), aeration tank water (E) and raw water (F) using STAMP at the genus level. The left panel shows the abundance proportion of different taxa in the two groups of samples. The middle panel represents the difference in abundance proportion of taxa within the 95% confidence interval. The value on the far right is the *P*-value; *P* < 0.05 indicates a significant difference and is marked in red (only the top 25 taxa with the lowest *P*-values are shown, at the genus level). Sludge (A: A1, A2, A3), sedimentation tank water (C: C1, C2, C3), aeration tank water (E: E1, E2, E3) and raw water (F: F1, F2, F3).

STAMP software was used to compare the taxa and functional abundance between different wastewater samples. A total of 25 KEGG metabolic pathways with significant differences (Welch’s t-test, adjusted *P* < 0.05) were obtained between bacterial samples of sludge and raw water (Fig. 5).

Predicted KEGG functions of sludge (A: A1, A2, A3), sedimentation tank water (C: C1, C2, C3), aeration tank water (E: E1, E2, E3) and raw water (F: F1, F2, F3) were classified and compared. The results showed that the pathway metabolism of cofactors and vitamins was predicted to be the most enriched in the comparison between A and C, with a significantly higher enrichment in group C than in group A. Similarly, this pathway was also predicted to be the most enriched in A *vs*. E, and the enrichment in group E was significantly higher than that in group A. In the comparison between A and F, Amino acid metabolism showed the highest enrichment, which was predicted to be significantly higher in group F than in group A. For C *vs*. E, metabolism of cofactors and vitamins was the most enriched pathway, with a higher enrichment in group E than in group C. In C *vs*. F, the most enriched pathway was also metabolism of cofactors and vitamins, and the enrichment in group F was higher than that in group C. In the comparison between E and F, carbohydrate metabolism was the most enriched pathway, with a significantly higher enrichment in group E than in group F.

**Figure 6:**
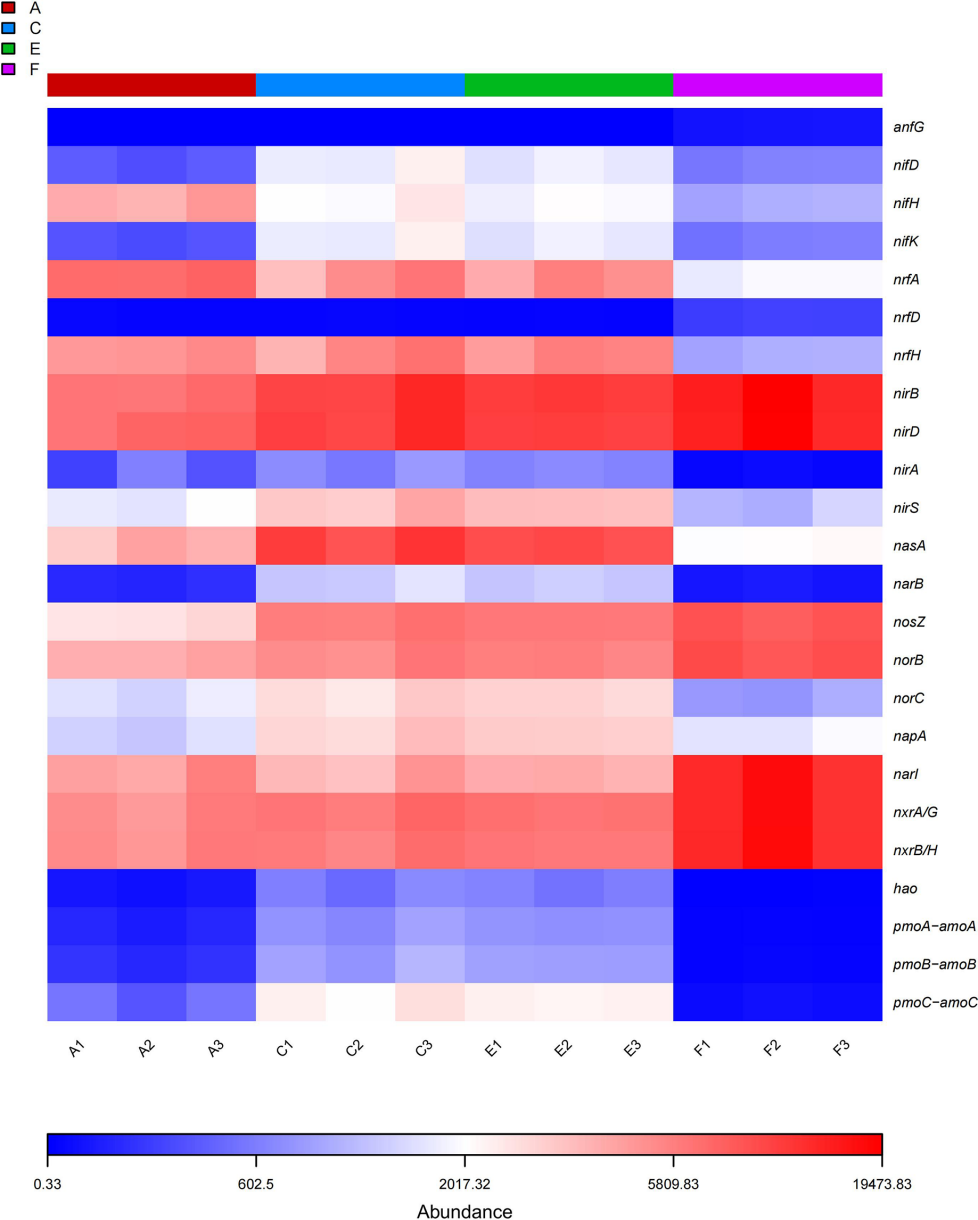
Abundance profiles of nitrogen metabolism-related functional genes in sludge (A), sedimentation tank water (C), aeration tank water (E) and raw water (F) samples. The horizontal axis represents the samples, and the vertical axis represents the abundance proportion of the guild in different samples. The vertical axis represents the relative abundance of nitrogen metabolism-related functional genes.

In addition, potential functional characteristics related to nitrogen metabolism were inferred in sludge (group A), sedimentation tank water (group C), aeration tank water (group E) and raw water (group F) samples from the wastewater treatment plant. A total of 24 predicted functional genes associated with nitrogen metabolism were inferred (Fig. 6, Additional file 7 Table S7), covering key nitrogen metabolism processes including nitrification, assimilatory nitrate reduction, dissimilatory nitrate reduction and nitrogen fixation. These results suggest that the microbial community in this wastewater treatment plant may possess the functional potential to participate in the nitrogen cycle. The presence of these predicted pathways may reflect the influence of untreated domestic sewage inputs, providing a functional gene-level reference for optimizing nitrogen removal efficiency.

#### COG functional annotation analysis

**Figure 7:**
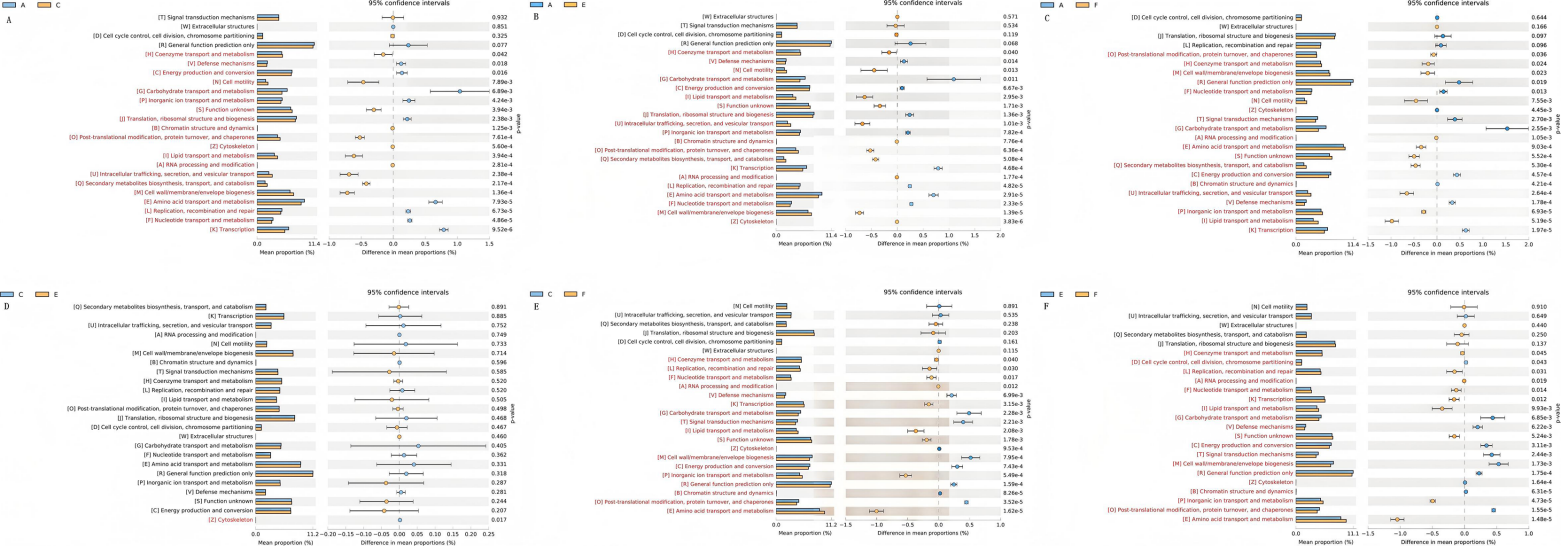
COG functional classification and enrichment analysis of microbial communities in sludge (A), sedimentation tank water (C), aeration tank water (E) and raw water (F). The left panel shows the abundance proportion of different taxa in the two groups of samples. The middle panel represents the difference in abundance proportion of taxa within the 95% confidence interval. The value on the far right is the *P* value; *P* < 0.05 indicates a significant difference and is marked in red (only the top 24 taxa with the lowest *P* values are shown, at the genus level). Sludge (A: A1, A2, A3), sedimentation tank water (C: C1, C2, C3), aeration tank water (E: E1, E2, E3) and raw water (F: F1, F2, F3).

The COG functions of sludge (A: A1, A2, A3), sedimentation tank water (C: C1, C2, C3), aeration tank water (E: E1, E2, E3) and raw water (F: F1, F2, F3) were classified and compared. The results showed that [R] general function prediction only was predicted to be the most enriched function in all pairwise comparisons, with a higher enrichment in group A than in group C and group E, and a significantly higher enrichment in group A than in group F; the enrichment was also higher in group C than in group E, significantly higher in group C than in group F, and significantly higher in group E than in group F (Fig. 7) (Welch’s t-test, adjusted *P* < 0.05).

#### Fungal functional prediction analysis

**Figure 8:**
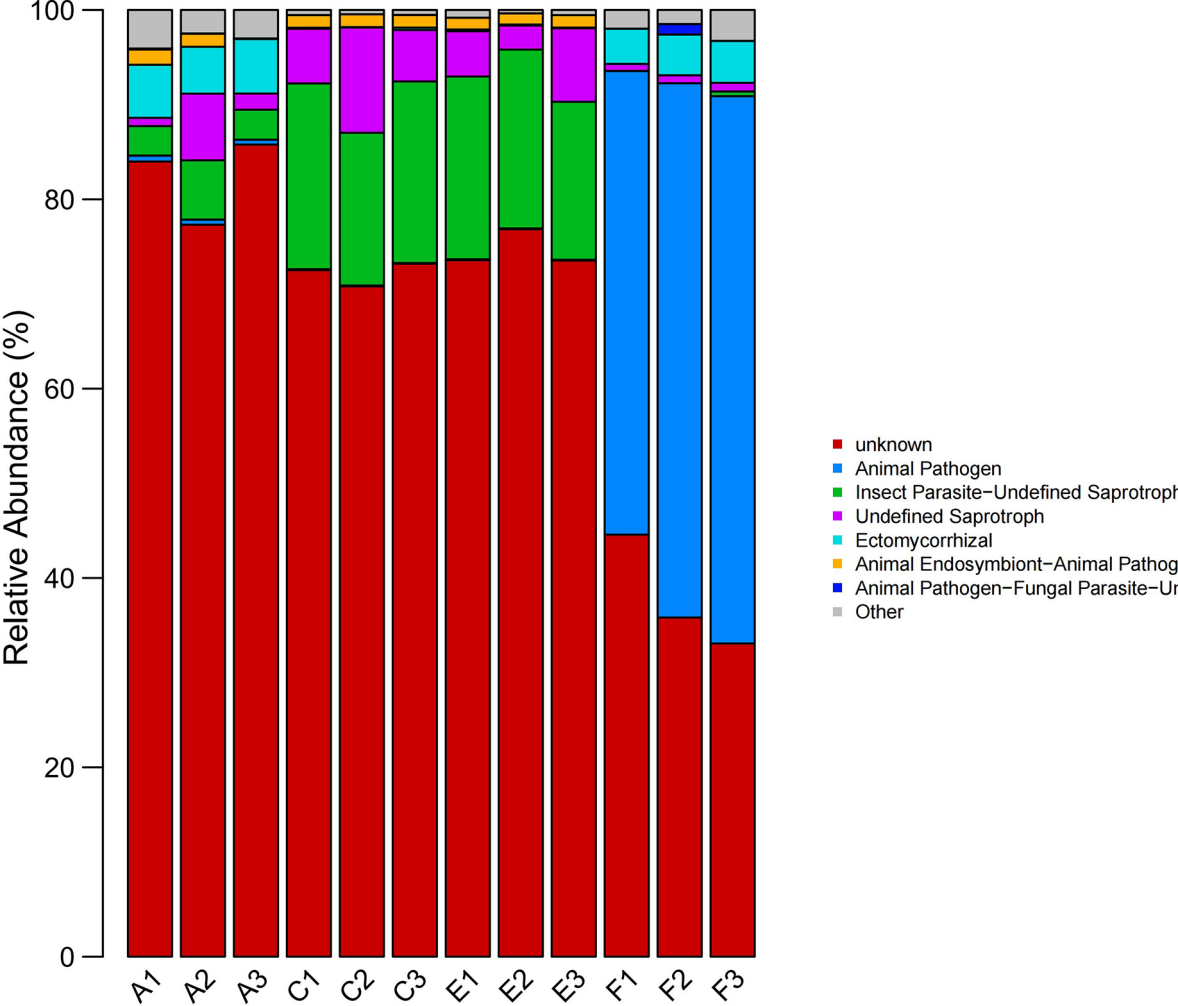
FUNGuild functional guild prediction and relative abundance analysis of fungal communities in sludge (A), sedimentation tank water (C), aeration tank water (E) and raw water (F). Rows represent unique functional guild categories, columns denote sample groups (sludge (A: A1, A2, A3), sedimentation tank water (C: C1, C2, C3), aeration tank water (E: E1, E2, E3) and raw water (F: F1, F2, F3)), and values indicate the relative abundance (%) of each guild in the corresponding sample.

FUNGuild functional prediction was conducted for fungi in sludge (A: A1, A2, A3), sedimentation tank water (C: C1, C2, C3), aeration tank water (E: E1, E2, E3) and raw water (F: F1, F2, F3). Based on Tukey’s honestly significant difference (HSD) test, distinct functional profiles were observed across the groups. In sludge, fungi were predicted to be dominated by Ectomycorrhizal (5.44 ± 0.43%) and Insect Parasite-Undefined Saprotroph (4.18 ± 1.80%), with Ectomycorrhizal being significantly higher in group A than in groups C, E, and F (adjusted *P* < 0.01), which suggests that fungi in this group are likely associated with unknown functions and plant symbiosis. Fungi in sedimentation tank water were assigned to Insect Parasite-Undefined Saprotroph (18.31 ± 1.90%) and Undefined Saprotroph (7.44 ± 3.19%), where Insect Parasite-Undefined Saprotroph showed no significant difference between groups C and E but was significantly higher in group E than in group F (adjusted *P* < 0.0001), implying their potentialhigher activity in insect parasitism and saprophytism. Fungi in aeration tank water were also assigned to Insect Parasite-Undefined Saprotroph (18.29 ± 1.14%), with a functional profile similar to that in sedimentation tank water, implying similar ecological roles. The predicted relative abundance of Animal Pathogen in raw water reached 54.41 ± 4.75%, and was significantly higher than that in groups A, C, and E (adjusted *P* < 0.0001), while the proportion of unknown fungi dropped markedly to 37.83 ± 6.01%, and the abundances of Ectomycorrhizal (4.14 ± 0.38%) and other saprotrophic groups were extremely low, suggesting that fungi in this group are likely dominated by animal pathogenic functions. Overall, fungal communities in raw water exhibited a distinct functional shift characterized by a significant enrichment of animal pathogenic fungi, whereas Insect Parasite-Undefined Saprotroph maintained high abundance in sludge, sedimentation tank water and aeration tank water, highlighting a clear functional divergence between the raw influent and the treated units (Fig. 8, Additional file 8 Table S8).

## Discussion

In this study, high-throughput sequencing and bioinformatics analysis were used to systematically dissect the community structure, diversity and functional differences of bacterial and fungal communities in different treatment units (sludge group A, sedimentation tank water group C, aeration tank water group E, raw water group F) of the wastewater treatment plant in Qian’an City. The results provide a key basis for understanding the microbial-driven mechanism of wastewater purification and process optimization, and can be discussed from three aspects: community differentiation characteristics, taxa-function relationships and practical application values (Wei et al., 2018; Wang et al., 2020; Yu, Tang & Lee, 2021). It should be noted that microbial identification was performed by a third-party company using standardized amplicon sequencing with the SILVA_v138.1 and UNITE_v9.0 databases (Quast et al., 2013; Abarenkov et al., 2024). Although 16S rRNA amplicon sequencing has been widely adopted and validated for environmental microbial community profiling (Caporaso et al., 2011), conventional short-read sequencing of hypervariable regions exhibits inherent limitations in species-level taxonomic resolution (Poretsky et al., 2014; Johnson et al., 2019). Therefore, future studies will introduce metagenomic sequencing or long-read full-length 16S rRNA sequencing technologies to further improve the accuracy and resolution of microbial taxonomic identification (Johnson et al., 2019).

From the perspective of microbial community diversity and structural differentiation, it is generally recognized that environmental heterogeneity of different treatment units serves as a key driver for microbial community differentiation (Huber et al., 2020). Alpha diversity analysis showed that bacterial and fungal richness were consistently lowest in raw water (group F). For bacteria, Chao and ACE indices in groups A, C and E showed no significant differences (adjusted *P* > 0.05), but were all significantly higher than those in group F (Chao: adjusted *P* < 0.0001; ACE: adjusted *P* < 0.01–0.05). Fungal richness followed a similar pattern, with groups C, E and A significantly richer than group F (adjusted *P* < 0.05), except for ACE between A and F (adjusted *P* > 0.05). Shannon, Simpson and Shannoneven indices also differed significantly between group A and the other groups (adjusted *P* < 0.0001). The high richness of both bacteria and fungi in the sludge unit (group A) is presumed to be related to its microenvironmental characteristics—this unit is presumed to be characterized by pollutant accumulation and sufficient nutrients, providing abundant metabolic substrates for microorganisms and promoting microbial proliferation and taxa coexistence (Sugurbekova et al., 2023; Gao et al., 2020). In contrast, raw water (group F) typically presents complex and fluctuating pollutant composition as well as poor environmental stability, which can only support the survival of a few stress-tolerant microorganisms, leading to a significant reduction in richness (Peng & Li, 2023; Leão et al., 2023). PCA analyses of β diversity further confirmed that the bacterial and fungal communities in groups C and E had extremely high similarity, but significant differences from groups A and F. The high similarity between groups C and E can be attributed to their shared position within the aeration-sedimentation treatment process. Consistent with the operational design, these units are expected to maintain similar process parameters such as dissolved oxygen levels and hydraulic retention time, thereby forming convergent microbial niches (Sun et al., 2020). However, the anaerobic/anoxic environment and high solid content of sludge (group A), as well as the extraneous microbial input in raw water (group F), collectively result in the microbial community differentiation between these two groups and the water treatment units, which is consistent with the coupling law of “process parameters—environmental factors—microbial community structure” in most wastewater treatment systems (Liu et al., 2020).

In terms of the functional adaptability of taxa composition, the dominant flora at the phylum and genus levels showed obvious unit-specificity and were highly compatible with the functional requirements of wastewater treatment. In the bacterial community, group A was significantly enriched by Chloroflexi (22.77 ± 4.77%) and Firmicutes (21.68 ± 2.31%). Chloroflexi are capable of degrading complex organic matter, and Firmicutes participate in organic acid fermentation under anaerobic conditions; the two phyla synergistically support organic matter degradation in the sludge unit (Bovio-Winkler, Cabezas & Etchebehere, 2024; Buckel, 2021; Giacon et al., 2022). Groups C and E were dominated by Proteobacteria and Bacteroidota. The genus *Thauera* in *Proteobacteria* (accounting for 5.79 ± 0.34% in group E) is a typical denitrifying bacterium that can efficiently convert nitrate to nitrogen gas. *Bacteroidota* excel at decomposing macromolecular organic matter such as proteins and polysaccharides, which well matches the needs of nitrification-denitrification nitrogen removal in the aeration tank and organic matter removal in the sedimentation tank (Zhao et al., 2013). In raw water (group F), Bacteroidota (40.24 ± 3.45%) and Proteobacteria (34.47 ± 3.62%) accounted for a high proportion, with enrichment of *Cloacibacterium* (36.35 ± 3.36%) and *Streptococcus* (14.39 ± 0.23%). The former is commonly found in polluted water to degrade small-molecule organic matter, while some strains of the latter are opportunistic pathogens, explaining the relatively high potential environmental risk of group F (Tan et al., 2024; Ye et al., 2016; Ramos-Sevillano et al., 2024). In the fungal community, group A was dominated by Rozellomycota (47.97 ± 7.08%), most of which are parasitic or symbiotic fungi that may form interactions with bacteria in sludge (Stüer-Patowsky et al., 2025). Groups C and E were overwhelmingly dominated by *Blastocladiomycota*, with no significant difference between them (adjusted *P* > 0.05), yet both were markedly higher than groups A and F. Within this phylum, the genus *Catenaria* is a dominant endoparasitic fungus of nematodes in activated sludge and has been detected in activated sludge from many wastewater treatment plants (Gray, 1984). In contrast, group F had the highest proportion of *Ascomycota* (61.40 ± 6.12%) and enrichment of *Simplicillium* (53.20 ± 4.09%), some strains of which are animal pathogens. Combined with the 54.41 ± 4.75% proportion of animal pathogenic fungi in group F, this indicates that raw water may carry biosafety risks, and disinfection should be strengthened in subsequent treatment (Hashimoto et al., 2021). Comparative analyses with other full-scale municipal WWTPs in China and internationally indicate that both bacterial and fungal community structures observed here largely conform to patterns reported across WWTPs. The dominance of Proteobacteria and Bacteroidota in aerobic and settling units, along with the enrichment of Chloroflexi and Firmicutes in sludge, is consistent with bacterial assembly rules described in multiple studies (Saunders et al., 2016; Zhang, Xu & Zhu, 2017; Wang et al., 2020). Similarly, the prevalence of saprotrophic and parasitic fungal guilds in activated sludge aligns with previous observations from both domestic and international plants (Wei et al., 2018). However, the exceptionally high proportion of animal pathogenic fungi in raw water (54.41 ± 4.75%) represents a distinctive feature that has rarely been reported in typical municipal WWTPs and more closely resembles systems receiving mixed industrial or livestock-derived wastewater. These cross-study comparisons suggest that, while the overall microbial assembly patterns are conserved among WWTPs, the unique taxonomic and functional traits identified in this study are primarily driven by local influent characteristics and process configuration.

Analysis of functional genes and metabolic pathways further suggests the wastewater treatment potential and optimization directions of the microbial community. PICRUSt2 prediction showed that there were 25 differential KEGG metabolic pathways between bacteria in group A and group C. Group A had a higher enrichment of carbohydrate metabolism pathways, which is consistent with the predicted organic matter degradation function of sludge, while group C was more active in cofactor and vitamin metabolism pathways, which could provide necessary coenzyme support for microbial nitrogen removal. More importantly, a total of 24 predicted functional genes related to nitrogen metabolism were inferred in this study, covering key processes such as nitrification, assimilatory/dissimilatory nitrate reduction, and nitrogen fixation, suggesting that the microbial community of the wastewater treatment plant may harbor the genetic potential for nitrogen cycling (Yu & Zhang, 2012). Additionally, group E (aeration tank) had a relatively high abundance of predicted denitrification functional genes, which may serve as genetic-level support for the system’s nitrogen removal efficiency. COG functional annotation indicated that group A was more prominent in predicted functions related to DNA replication, repair, and environmental adaptation, implying the adaptive strategies of sludge microorganisms to complex environments. Group F showed a higher proportion in [E] Amino acid transport and metabolism (9.79% *vs*. 9.45% in group A), which was consistent with its need to cope with complex pollutants in raw water. FUNGuild analysis for fungal function prediction indicated that groups A, C, and E were primarily assigned to unknown functions (72.18~82.36%) and Insect Parasite-Undefined Saprotroph (4.18~18.31%), while the predicted proportion of animal pathogenic fungi in group F exceeded half. This difference suggests that attention should be paid to pathogenic microorganism control in the raw water pretreatment process to prevent them from entering subsequent treatment units and affecting the safety of effluent water.

From a practical application perspective, the results of this study provide clear targets for process optimization of the wastewater treatment plant in Qian’an City. To improve nitrogen removal efficiency, based on the enrichment characteristics of denitrifying bacteria such as Thauera in group E, the dissolved oxygen gradient in the aeration tank can be optimized while an appropriate supplement of carbon sources is applied to promote the denitrification process. For the sludge unit (group A), the high abundance of Chloroflexi and Firmicutes suggests that adjusting the sludge retention time (SRT) can extend the degradation period of complex organic matter and enhance sludge reduction efficiency. Given the low microbial richness and high pathogenic risk in raw water (group F), it is recommended to add pretreatment units (such as grids, regulating tanks, and hydrolytic acidification tanks) to reduce pollutant fluctuations, and strengthen ultraviolet or chlorine disinfection at the effluent end to control the discharge of pathogenic fungi. In addition, the enrichment of Cloacibacterium and Streptococcus in group F, as well as the high abundance of norank_Anaerolineaceae (14.14 ± 2.74%) in group A, can serve as potential indicator microorganisms for microbial monitoring in wastewater treatment systems; by tracking the abundance changes of these representative flora, the operation status of each unit can be evaluated in real time, providing rapid feedback for process regulation.

It should be noted that this study failed to reflect the impact of seasonal changes on microbial community structure; future research could conduct multi-time series monitoring to analyze the dynamic regulatory effects of factors such as temperature and influent load. Additionally, the statistical framework treated units as independent groups, which may not fully capture the temporal autocorrelation along the treatment train. Moreover, although PCA plots visually demonstrated community separation, formal statistical tests for β diversity (*e.g*., permutational multivariate analysis of variance (PERMANOVA)) were not performed in this study. Meanwhile, functional prediction results need to be further verified through multi-omics technologies (*e.g*., metagenomics and metatranscriptomics) and microbial pure culture experiments to more accurately reveal the metabolic mechanisms of key functional microbial communities. Overall, this study clarified the distribution patterns and functional potential of the microbial community in the Qian’an Wastewater Treatment Plant, which not only fills the gap in microbial ecology research of this specific plant but also provides theoretical references for microbial regulation and process upgrading of similar wastewater treatment systems.

## Conclusion

This study analyzed the microbial diversity and predicted function of sludge (group A), sedimentation tank water (group C), aeration tank water (group E) and raw water (group F) samples from the wastewater treatment plant in Qian’an, and clarified the microbial characteristics and functional differences among different treatment units. Bacterial richness was highest in groups A, C, and E and lowest in group F, whereas fungal richness was highest in groups C and E and lowest in group F. Overall, groups C and E exhibited highly similar richness and diversity patterns, which differed markedly from those of groups A and F. At the phylum level, bacteria in group A were significantly enriched by Chloroflexi and Firmicutes, while groups C, E, and F were mainly composed of Proteobacteria and Bacteroidota, with Bacteroidota being most abundant in group F; fungi in group F were dominated by Ascomycota, and the dominant microbial communities at the genus level also showed obvious unit specificity. At the functional level, 25 differential KEGG metabolic pathways were observed between bacteria in groups A and C, and a total of 24 predicted functional genes related to nitrogen metabolism were inferred, suggesting the presence of nitrogen cycle potential within the microbial community of this wastewater treatment plant. Meanwhile, the predicted relative abundance of animal pathogenic fungi in group F was as high as 54.41 ± 4.75%, which was significantly different from that in other units. The results of this study provide a scientific basis for optimizing the wastewater treatment process and provide theoretical guidance for enhancing nitrogen removal efficiency of the plant, and also provide theoretical support for microbial regulation and process upgrading in wastewater treatment plants of similar food enterprises.

## Supplemental Information

10.7717/peerj.21546/supp-1 Supplemental Information 1 Raw data of bacterial community α-diversity indices (Shannon, Simpson, Chao, ACE) in sludge, sedimentation tank water, aeration tank water, and raw water of the wastewater treatment plant.

10.7717/peerj.21546/supp-2 Supplemental Information 2 Raw data of fungal community α-diversity indices (Shannon, Simpson, Chao, ACE) in sludge, sedimentation tank water, aeration tank water, and raw water of the wastewater treatment plant.

10.7717/peerj.21546/supp-3 Supplemental Information 3 Raw data of bacterial community relative abundance at the phylum level in different treatment units (sludge, sedimentation tank water, aeration tank water, raw water) of the wastewater treatment plant.

10.7717/peerj.21546/supp-4 Supplemental Information 4 Raw data of fungal community relative abundance at the phylum level in different treatment units (sludge, sedimentation tank water, aeration tank water, raw water) of the wastewater treatment plant.

10.7717/peerj.21546/supp-5 Supplemental Information 5 Raw data of bacterial community relative abundance at the genus level in different treatment units (sludge, sedimentation tank water, aeration tank water, raw water) of the wastewater treatment plant.

10.7717/peerj.21546/supp-6 Supplemental Information 6 Raw data of fungal community relative abundance at the genus level in different treatment units (sludge, sedimentation tank water, aeration tank water, raw water) of the wastewater treatment plant.

10.7717/peerj.21546/supp-7 Supplemental Information 7 Raw data of bacterial community PICRUSt2 functional prediction (focusing on nitrogen metabolism-related functional genes) in sludge, sedimentation tank water, aeration tank water, and raw water of the wastewater treatment plant.

10.7717/peerj.21546/supp-8 Supplemental Information 8 Raw data of fungal functional prediction in different treatment units (sludge, sedimentation tank water, aeration tank water, raw water) of the wastewater treatment plant.

10.7717/peerj.21546/supp-9 Supplemental Information 9 Combined statistics of sequencing data processing results. Combined statistics of sequencing data processing results for bacterial and fungal samples from sludge, sedimentation tank water, aeration tank water and raw water of a wastewater treatment plant, including sequence number, mean length, minimum length and maximum length

## Additional Information and Declarations

### DNA Deposition

The following information was supplied regarding the deposition of DNA sequences:

The raw sequence data are available at (NMDC): NMDC40095867–NMDC40095890.

### Data Availability

The following information was supplied regarding data availability:

The data and code are available at GitHub:

- https://github.com/wch/r-source/blob/trunk/src/library/graphics/R/barplot.R.

- https://github.com/vegandevs/vegan.

- https://github.com/emmanuelparadis/ape.

- https://github.com/kassambara/ggpubr.

The raw sequence data are available at (NMDC): NMDC40095867–NMDC40095890.
