# Supplementary material for: Microbial diversity and functional analysis in wastewater and sludge of wastewater treatment plants
Source: PeerJ. 2026 Jul 16;14:e21546. doi: 10.7717/peerj.21546 (PMC13380884; doi:10.7717/peerj.21546)
Supplement: Supplemental Information 9 — Combined statistics of sequencing data processing results for bacterial and fungal samples from sludge, sedimentation tank water, aeration tank water and raw water of a wastewater treatment plant, including sequence number, mean length, minimum length and maximum length [file peerj-14-21546-s009.docx]

**Microbial diversity and functional analysis in wastewater and sludge of wastewater treatment plants**

Yazi Li^1^, Shuhong Zhang^1^, Ke Xu^1^, Jingjie Zhang^1^, Man Dong^2^, Huan Lu^3^, Yongshan Fan^1*^

1. Department of Life Sciences, Hebei Key Laboratory of Plant Biotechnology Research and Application, Tangshan Normal University, Tangshan, Hebei, China.

2. College of Plant Protection, Yunnan Agricultural University, Kunming, Yunnan, China.

3. College of Life Sciences, Agricultural University of Hebei, Baoding, Hebei, China.

*Corresponding Author:

Yongshan Fan^1^

Tangshan, Hebei 063000, China

Email address: fanyongshan@126.com

**This file includes:**

Supplementary Table 1-3

**Supplementary Table 1 Sample metadata and NMDC sequence accession numbers**

| Sample Group | Replicate ID (Figure) | Sample Type | Target Gene | NMDC Accession Number |
| --- | --- | --- | --- | --- |
| A | A1 | Sludge | 16S rRNA (Bacteria) | NMDC40095867 |
|  | A2 | Sludge | 16S rRNA (Bacteria) | NMDC40095868 |
|  | A3 | Sludge | 16S rRNA (Bacteria) | NMDC40095869 |
|  | A1 | Sludge | ITS (Fungi) | NMDC40095870 |
|  | A2 | Sludge | ITS (Fungi) | NMDC40095871 |
|  | A3 | Sludge | ITS (Fungi) | NMDC40095872 |
| C | C1 | Sedimentation tank water | 16S rRNA (Bacteria) | NMDC40095873 |
|  | C2 | Sedimentation tank water | 16S rRNA (Bacteria) | NMDC40095874 |
|  | C3 | Sedimentation tank water | 16S rRNA (Bacteria) | NMDC40095875 |
|  | C1 | Sedimentation tank water | ITS (Fungi) | NMDC40095876 |
|  | C2 | Sedimentation tank water | ITS (Fungi) | NMDC40095877 |
|  | C3 | Sedimentation tank water | ITS (Fungi) | NMDC40095878 |
| E | E1 | Aeration tank water | 16S rRNA (Bacteria) | NMDC40095879 |
|  | E2 | Aeration tank water | 16S rRNA (Bacteria) | NMDC40095880 |
|  | E3 | Aeration tank water | 16S rRNA (Bacteria) | NMDC40095881 |
|  | E1 | Aeration tank water | ITS (Fungi) | NMDC40095882 |
|  | E2 | Aeration tank water | ITS (Fungi) | NMDC40095883 |
|  | E3 | Aeration tank water | ITS (Fungi) | NMDC40095884 |
| F | F1 | Raw water | 16S rRNA (Bacteria) | NMDC40095885 |
|  | F2 | Raw water | 16S rRNA (Bacteria) | NMDC40095886 |
|  | F3 | Raw water | 16S rRNA (Bacteria) | NMDC40095887 |
|  | F1 | Raw water | ITS (Fungi) | NMDC40095888 |
|  | F2 | Raw water | ITS (Fungi) | NMDC40095889 |
|  | F3 | Raw water | ITS (Fungi) | NMDC40095890 |

Note: Paired bacterial and fungal samples were collected from the same treatment units. Fungal samples were assigned the same figure labels as their paired bacterial samples (e.g., fungal A1 corresponds to bacterial A1 from the same sludge sample) to indicate they originated from the same physical material. Due to database constraints preventing duplicate sample names, these fungal samples were submitted to NMDC with adjusted identifiers (e.g., A4–A6 for sludge fungi, where A4 corresponds to figure label A1). These submitted labels differ from the figure labels used in this manuscript, but both refer to the same set of samples.

**Supplementary Table 2** **Statistics of sequencing data processing results for bacterial samples from wastewater treatment plant**

| Group | Sample | SeqNum | MeanLen | MinLen | MaxLen |
| --- | --- | --- | --- | --- | --- |
| A | A1 | 61691 | 416.36 | 352 | 455 |
| A | A2 | 68007 | 415.1 | 350 | 459 |
| A | A3 | 69956 | 416.26 | 351 | 460 |
| C | C1 | 56056 | 419.41 | 350 | 460 |
| C | C2 | 57938 | 420.29 | 352 | 461 |
| C | C3 | 76433 | 419.96 | 352 | 467 |
| E | E1 | 58063 | 420.32 | 350 | 467 |
| E | E2 | 60554 | 419.97 | 350 | 458 |
| E | E3 | 58409 | 420.5 | 350 | 460 |
| F | F1 | 62748 | 424.14 | 350 | 436 |
| F | F2 | 63663 | 424.44 | 351 | 452 |
| F | F3 | 60364 | 424.2 | 354 | 434 |

Note: Sludge (A: A1, A2, A3), Sedimentation tank water (C: C1, C2, C3), Aeration tank water (E: E1, E2, E3) and Raw water (F: F1, F2, F3)

**Supplementary Table 3 Statistics of sequencing data processing results for fungal samples from wastewater treatment plant**

| Group | Sample | SeqNum | MeanLen | MinLen | MaxLen |
| --- | --- | --- | --- | --- | --- |
| A | A1 | 135809 | 221.64 | 102 | 451 |
| A | A2 | 122982 | 223.61 | 100 | 449 |
| A | A3 | 184406 | 223.86 | 101 | 451 |
| C | C1 | 136935 | 241.11 | 102 | 446 |
| C | C2 | 110456 | 240.34 | 107 | 443 |
| C | C3 | 115587 | 241.01 | 102 | 446 |
| E | E1 | 119227 | 243.68 | 102 | 451 |
| E | E2 | 124731 | 240.03 | 102 | 450 |
| E | E3 | 124081 | 240.7 | 108 | 447 |
| F | F1 | 151814 | 250.23 | 100 | 451 |
| F | F2 | 158438 | 244.72 | 101 | 451 |
| F | F3 | 126631 | 249.51 | 101 | 451 |

Note: Sludge (A: A1, A2, A3), sedimentation tank water (C: C1, C2, C3), aeration tank water (E: E1, E2, E3) and raw water (F: F1, F2, F3).

**
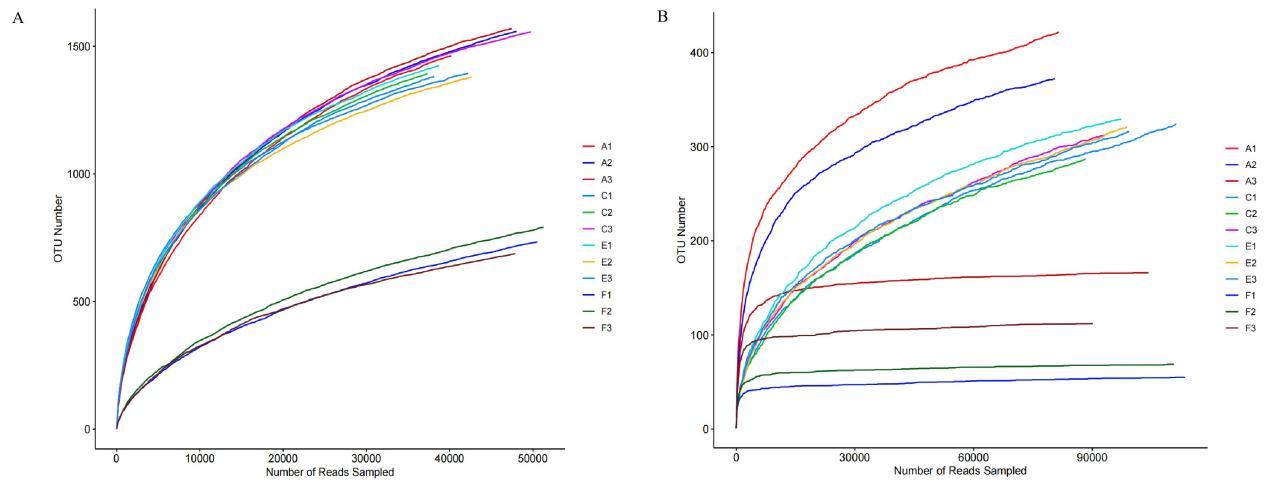
**

**Supplementary Figure S1 Rarefaction curves of alpha indices for bacterial and fungal samples from wastewater treatment plant.**

Sludge (A: A1, A2, A3), sedimentation tank water (C: C1, C2, C3), aeration tank water (E: E1, E2, E3) and raw water (F: F1, F2, F3). The horizontal axis represents the number of randomly sampled sequences in each sample, and the vertical axis represents the corresponding α index. Each curve stands for one sample (taking the number of OTUs as an example).
